# Increased future occurrences of the exceptional 2018–2019 Central European drought under global warming

**DOI:** 10.1038/s41598-020-68872-9

**Published:** 2020-08-06

**Authors:** Vittal Hari, Oldrich Rakovec, Yannis Markonis, Martin Hanel, Rohini Kumar

**Affiliations:** 1 UFZ-Helmholtz Centre for Environmental Research, 04318 Leipzig, Germany; 2 Faculty of Environmental Sciences, Czech University of Life Sciences Prague, Kamýcká 129, 165 00 Praha-Suchdol, Czech Republic

**Keywords:** Climate sciences, Hydrology

## Abstract

Since the spring 2018, a large part of Europe has been in the midst of a record-setting drought. Using long-term observations, we demonstrate that the occurrence of the 2018–2019 (consecutive) summer drought is unprecedented in the last 250 years, and its combined impact on the growing season vegetation activities is stronger compared to the 2003 European drought. Using a suite of climate model simulation outputs, we underpin the role of anthropogenic warming on exacerbating the future risk of such a consecutive drought event. Under the highest Representative Concentration Pathway, (RCP 8.5), we notice a seven-fold increase in the occurrence of the consecutive droughts, with additional 40 ($\pm \, 5$) million ha of cultivated areas being affected by such droughts, during the second half of the twenty-first century. The occurrence is significantly reduced under low and medium scenarios (RCP 2.6 and RCP 4.5), suggesting that an effective mitigation strategy could aid in reducing the risk of future consecutive droughts.

## Introduction

Human-induced climate change is evident and it poses a great concern to society, primarily due to its potential to intensify extreme events around the globe^1,2^. In the past 2 decades, Europe experienced an increased frequency of droughts^3,4^ with estimated loss of about EUR 100 billion^5^. One such devastating event was the drought in summer 2003, which was an exceptionally warm and dry year across most of central and western Europe. Historical reconstructions since 1500 C.E. suggest that it was one of the hottest summers^6^, and the event was estimated to result in a 30% reduction in gross primary production compared to previous years between 1998–2002^3^. Although, the 2003 drought event was rare and exceptional, even in a multi-centennial time window, its likelihood is expected to increase in the near future^7^, mainly due to the anthropogenic warming^8–11^.

**Figure 1:**
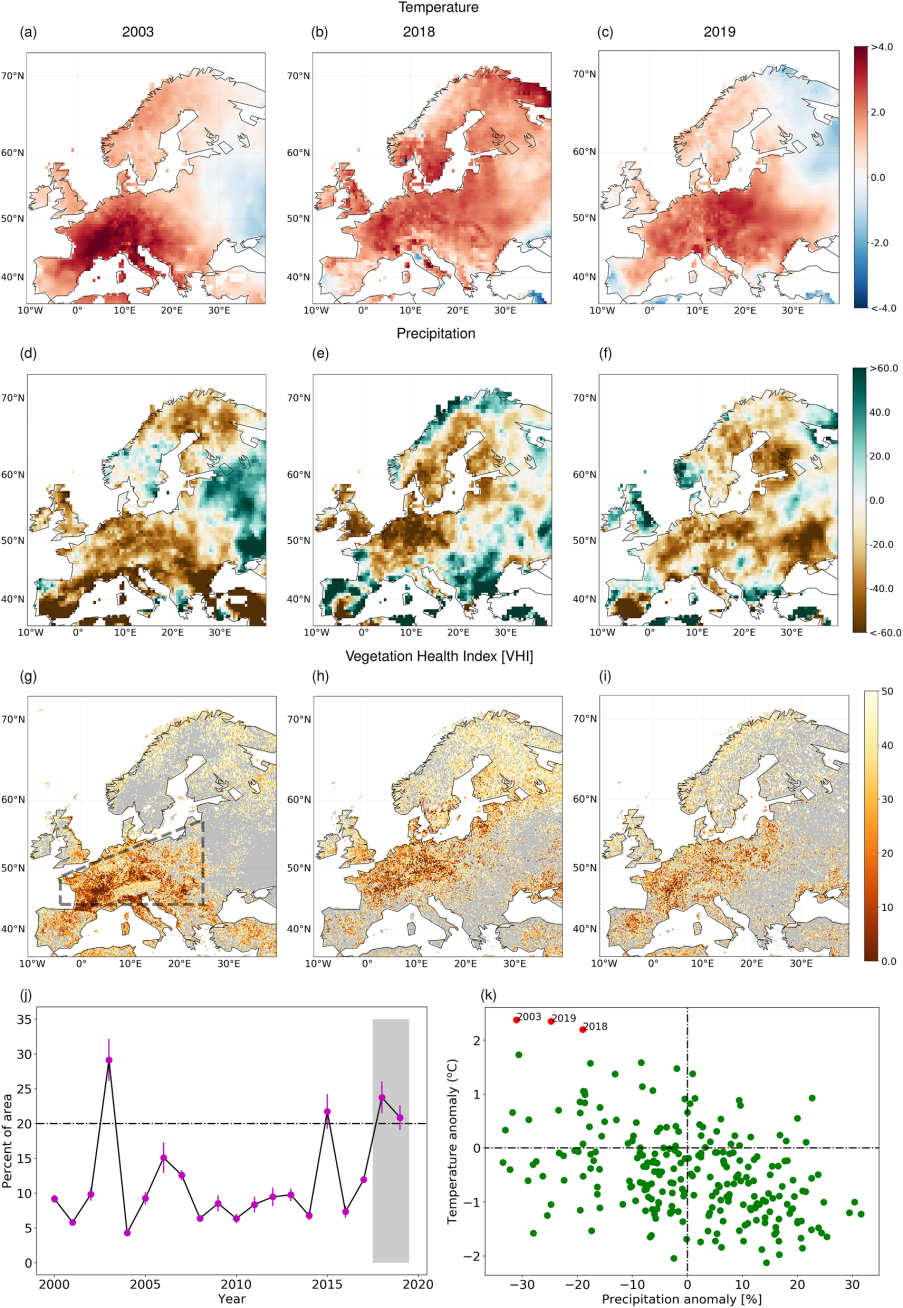
Anomalies of climate and vegetation health index (VHI) during 2003, 2018 and 2019. (**a**,**b**,**c**) Mean summer (June–August) temperature anomalies ($^\circ \hbox {C}$) for 2003, 2018 and 2019 based on the 1980–2010 climatology, and (**d**,**e**,**f**) their corresponding precipitation anomalies (%). (**g**,**h**,**i**) Vegetation condition in terms of VHI during 2003, 2018 and 2019, respectively. (**j**) Yearly development of the summer time, percentage area with poor vegetation health (i.e., VHI $\le 30$) estimated over the Central European region (depicted by a black rectangular region in the panel **g**) during the period 2000–2019. The thick black line shows the year-wise weekly mean of VHI during summer months, and the pink bar represents the corresponding 95% confidence limit based on the sampling distribution of the mean. The years 2003, 2015, 2018 and 2019 experienced the deprivation in the vegetation health, where the poor vegetation health extends over more than 20% of the central European region. The gray shaded region highlights the years 2018 and 2019, during which the poor vegetation health persists over more than 20% of the central European area, consecutively in 2 years. (k) Yearly summer-time precipitation and temperature anomalies estimated over the central Europe region during the 254 years. Three exceptional years of 2003, 2018 and 2019 are shown by the red dots, where the mean summer temperature anomalies over the Central Europe reached the record extreme conditions of more than $2\,^\circ \hbox {C}$; and precipitation anomalies show deficit of more than 20%. The maps in the figure are generated using Python version 3.7.3 (https://www.python.org/search/?q=Python+3.7.3).

In the summer of 2018, temperature anomaly broke the record again in several locations across Europe, but with distinct spatial patterns. While in summer 2003 the increase in temperature was more concentrated in central and southern Europe (Fig. 1a), summer 2018 was characterised by an anomalous increase in central and north-eastern Europe (Fig. 1b). Unlike the 2003 event—where the temperature anomaly (Supplementary Fig. S1) and the ecosystem carbon and energy fluxes recovered early after the summer^12^, the extreme event of 2018 persisted to the subsequent year 2019 (Fig. 1c). For all these years, the impact was strongest in the Central European region, where the increase in temperature was accompanied by concurrent significant reduction of summer precipitation (Fig. 1d–f), which led to extreme drought conditions.

The intensity and spatial extent of droughts significantly affects the plant and agricultural productivity^13,14^, underlying the severity of the drought impact in Central European region, where the focus on agriculture is strong^3,7,15–17^. With the use of remote sensing data-sets^18^, we find that the concurrent increased temperature with deficit precipitation impaired the condition for vegetation activities (Fig. 1g) in the summer of 2003. We show this in terms of vegetation health index (VHI), which represents the vegetation stress due to the droughts (see methods for detailed description). Similar observations have been made during the summer 2018 as well (Fig. 1h), when several countries suffered agro-economic shocks^19^. Further, the deprivation of vegetation health persisted and it is noticed even during the summer 2019 (Fig. 1i). In the case of the exceptional 2003 and 2015 events^4,20^, the vegetation health recovered and returned to its normal condition during the following years. On the contrary, the impact of the 2018 drought on vegetation activities propagated to 2019 and the recovery is still underway, as shown in the time series of VHI (Fig. 1j). Additionally, we note that the vegetation health being categorised in poor condition for at-least 20% of the Central European area in both 2018 and 2019 is unprecedented from the observations in the previous years of twenty-first century. Thus, it is with the utmost urgency that we need to recognise the importance of these persevering consecutive year events, and to develop a holistic framework to model the risk^21^.

## Results

### 2018–2019 Central European drought from the long-term observational records

**Figure 2:**
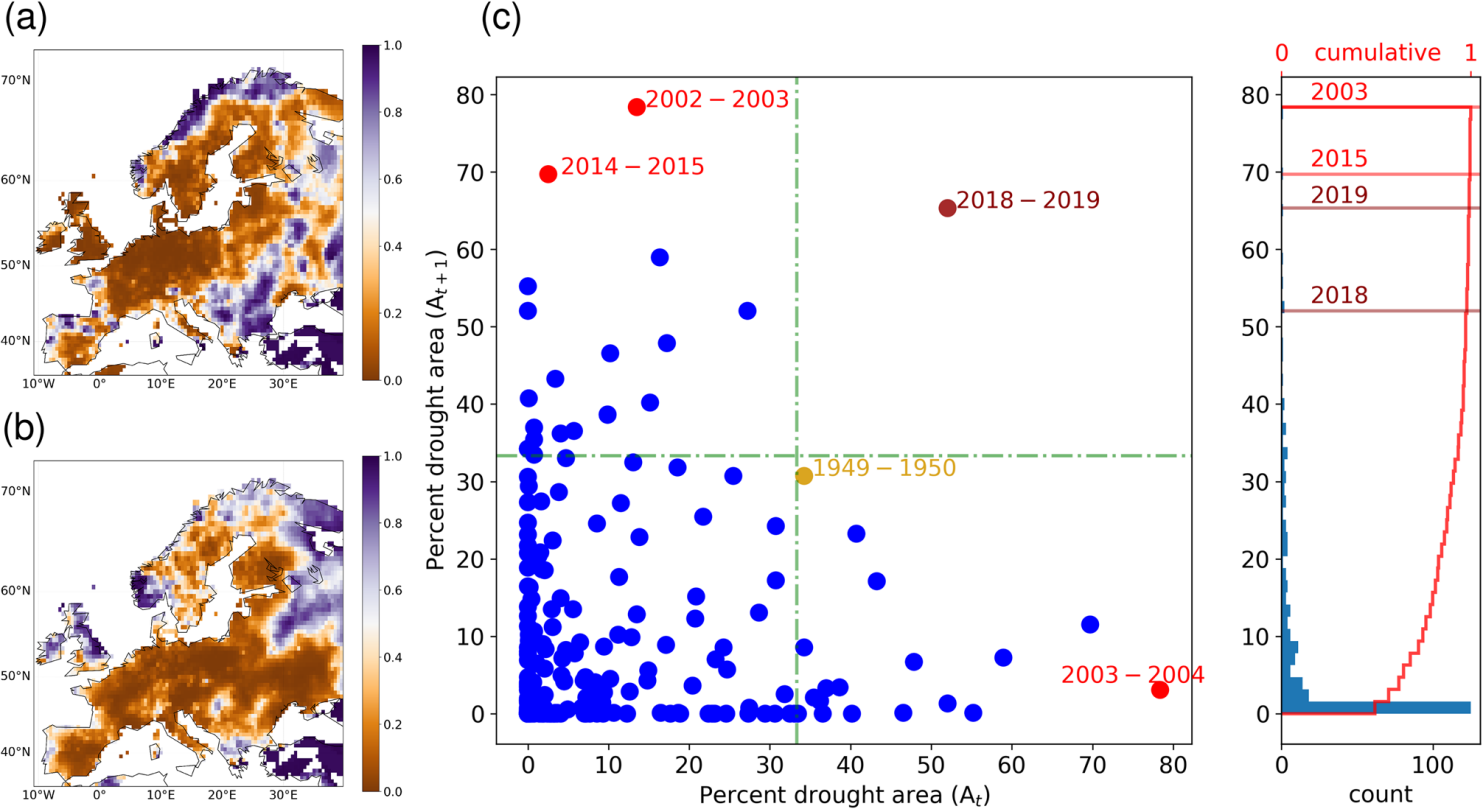
2-year droughts from the long-term observational records over the Central Europe. (**a**,**b**) Spatial distribution of the drought index estimated based on summer months (June–August) SPEI for 2018 and 2019. (**c**) Scatter plot showing the percent drought area over the Central Europe for the next year ($A_{t+1}$) as a function of current year drought area ($A_t$). Prominent drought years, viz., 1949, 1950, 2003, 2015; and the recent 2018 and 2019 years, during which the spatial extent of summer droughts are significantly higher than the rest are highlighted in red dots. The cumulative distribution of the percent drought area is shown in the right panel of (**c**), with highlighted major drought years. The green dashed lines in (**c**) depict the drought area threshold of 33.3%—i.e., one third of the Central Europe region. The 2018–2019 event stands alone as an exceptional event for the consecutive droughts during the last 254 years (1766–2019). The maps in the figure are generated using Python version 3.7.3 (https://www.python.org/search/?q=Python+3.7.3).

The historical reconstruction of composite 254-year long-term climatic database^22,23^ indicates that although the precipitation anomaly exhibits a drier than average situation during the summer months of 2018 and 2019 across the Central Europe, its intensity is not that high and there are also many other years with similar range of precipitation anomalies (Fig. 1k). On the other hand, 2018–2019 were two out of the three warmest summer periods in the record. To account for this joint effect of precipitation and temperature anomalies, we estimate the drought index based on the standardised precipitation evapotranspiration index (SPEI)^24^ that considers the atmospheric water supply and demand (see Methods). Our analysis further demonstrates the usefulness of the SPEI estimates as relevant climate predictors for characterising the temporal variability of the summer-time vegetation activities (see Supplementary Fig. S2). While the spatial pattern of summer 2018 SPEI (Fig. 2a) depicts severe drought conditions in the Central European region (SPEI $\le 0.1$; see Methods), southern Europe (Balkan countries) experienced wetter than normal conditions (i.e., SPEI $\ge 0.5$). Similar to 2018, a severe drought condition (SPEI $\le 0.1$) was noticed during the summer of 2019 but the spatial extent of drought was substantially larger compared to the 2018 event (Fig. 2b).

In Central Europe, over 34% of the total land area is extensively used for agricultural purposes^19^. Our analysis suggests that more than 50% of the Central European region suffered severe drought conditions in the consecutive years of 2018 and 2019. To examine how frequently these consecutive extreme events have occurred in the long-term observational records, we computed and plotted the areal extent of drought ($A_t$) with SPEI(*t*) $\le 0.1$ for a given year (*t*) with the corresponding estimates for the next year ($A_{t+1}$) (Fig. 2c). It is evident from the analysis that the 2018–2019 drought is a record breaking event in terms of the consecutive event in the last 254 years, with nearly 50% of the Central European area being classified under the extreme drought conditions. It is also worth mentioning that the 1949–1950 years ranked the second most large-scale consecutive drought years^25^. Nonetheless, in this case the spatial extent was considerably smaller (around 33%) than that of the 2018–2019 droughts.

The large-scale atmospheric circulation during 2018–2019 was characterized by pronounced positive geopotential height anomalies and anticyclonic circulation pattern at 500 hpa, covering a large area centered over Central Europe and extending to the Northern European region (Supplementary Fig. S3). The complex evolution of these blocking conditions highlights its contribution to the exceptional observed temperature anomalies during 2018–2019. Further, the persistent occurrence of these atmospheric blocking conditions are responsible for the development of large-scale droughts and heat wave, and also triggers soil-moisture temperature feedbacks^6,26–28^, which could further exacerbate and prolong concurrent soil drought and atmospheric aridity^29^. Literature review suggests that the recent arctic warming is likely to be a main driving factor causing more frequent extreme weather events across the mid-latitudes regions in the Northern Hemisphere^30–33^. The major dynamical features for changing the mid-latitude weather due to arctic amplification is the position and structure of the jet stream and planetary wave activity. Jet streams are primarily driven by the difference in temperature between the polar and mid-latitudinal regions. However, the reduced temperature gradient between these two regions has been suggested to lead to a weaker zonal jet with larger meanders^32^ and that this would cause weather systems to travel eastward rather slowly leading to more persistent weather patterns^34^. These movement activities are further going to be affected (more persistent) under future warming conditions with increased greenhouse emission^35,36^. Nevertheless, these theories/mechanisms are still being explored and debatable; and require further rigorous testing^37^. Moreover, in this study we restrict our focus on analysing (detecting) the exceptional 2018–2019 Central European drought from the long-term observational record point of view, and on the nature of their possible occurrences under warming worlds. Addressing the mechanism (attribution) of the 2018–2019 drought event itself is another line of research, which requires a comprehensive analysis and is beyond the scope of the present study.

### Future occurrences of 2-year droughts under global warming

**Figure 3:**
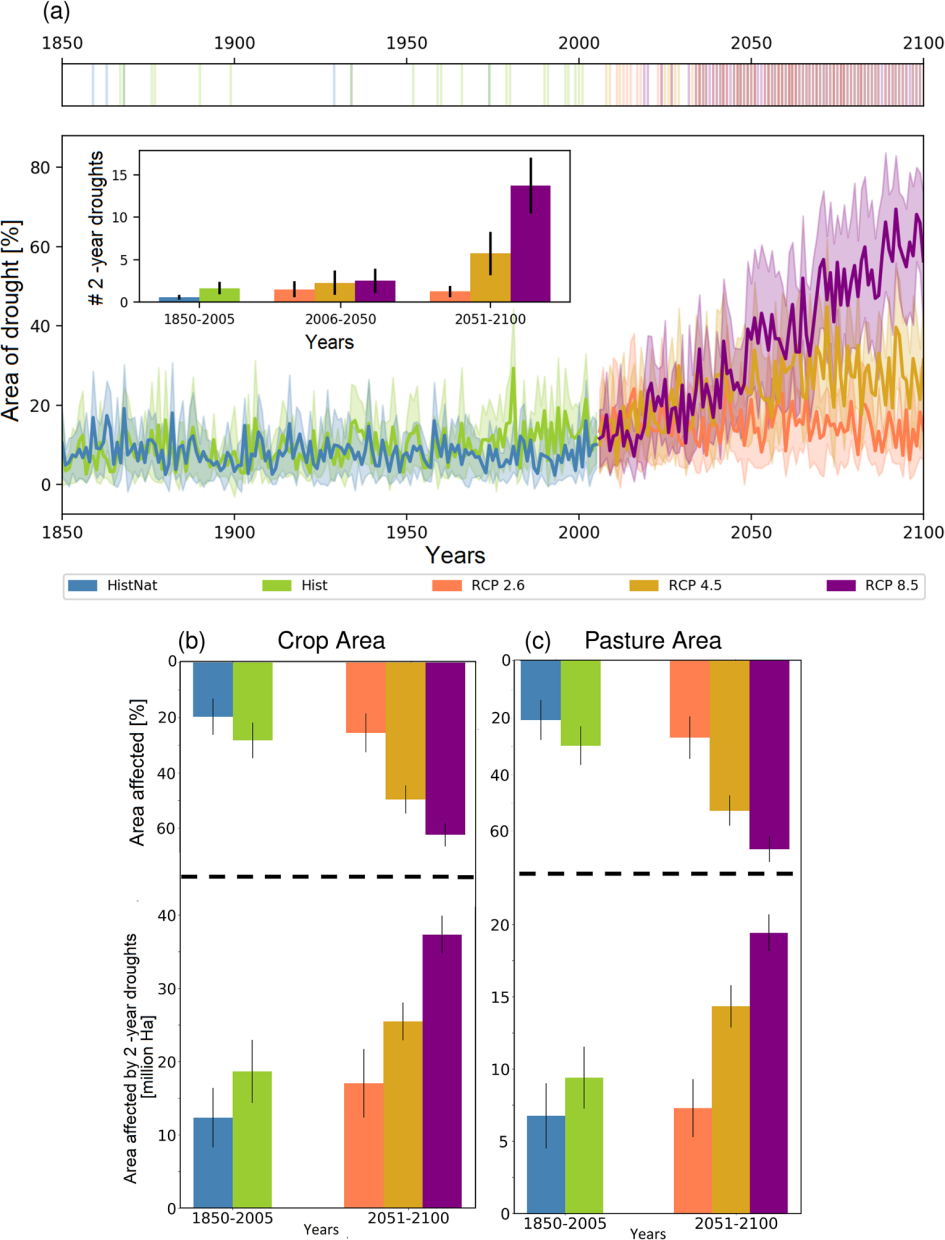
2-year droughts from state-of-the-art climate model simulations and its implications on cropland and pasture. (**a**) Yearly development of the percent area of drought over the Central Europe based on the ensemble ($\hbox {N} = 11$) of climate model simulations from CMIP5 under different experimental scenarios: natural only historical (HistNat), all-forcings historical (Hist), and three future RCPs (2.6, 4.5, and 8.5). The thick solid lines show the multimodel means, and the filled areas represent the 95% confidence intervals based on the sampling distribution of the mean from 11 GCMs simulations. The inset plot in (**a**) represents the number of 2-year droughts, with an areal extent in each year covering at-least one third of the Central European region, estimated over the specified time-period for different experimental scenarios (i.e., 1850–2005 for the Hist and HistNat; and 2006–2050/2051–2100 for the RCPs). Shown are the ensemble mean and 95% confidence limits based on the sampling distribution of the mean, corresponding to the 11 climate model outputs. The top panel of (**a**) depicts the year in which any of the 11 climate models show the 2-year droughts. The bottom panel of (**b**) shows cropland area (in million hectares) affected by the consecutive droughts under different experimental scenarios. The top panel of (**b**) shows the corresponding estimates in terms of percent of total cropland areas over the Central Europe, affected by the 2-year droughts. (**c**) Same as (**b**), but for pasture lands. The colors and ensemble statistics (i.e, mean and confidence intervals) are estimated as mentioned above.

From our observations, it is clear that, 2018–2019 is an unprecedented 2-year drought event. We now use the ensemble of climate model simulations from the Coupled Model Intercomparison Project phase 5 (CMIP5)^38^ (Table S1) to understand how the frequency of the 2-year drought event would change in the coming decades, and underpin the role of the anthropogenic warming in exacerbating such drought events (see Methods). In comparison to the simulations based on natural-only forced simulations (HistNat), the occurrence of the 2-year drought event shows a slight increase in the historical simulations (Hist) during the common period of 1850–2005 (Fig. 3a). The differences in the temporal evolution of areas of Central Europe affected by drought among the two sets of simulations have become more apparent during the last 30 years (approx. post 1970; Supplementary Fig. S4)—the period in which there are apparent indications of the role of anthropogenic activities exacerbating global warming^39^. Climate model simulations based on the Representative Concentration Pathway (RCP) 8.5 scenario further indicates a strong increase in areas under drought towards the end of the twenty-first century (Fig. 3a). Under a moderate scenario of RCP 4.5, the increasing trend persists until the middle of the twenty-first century and stagnates thereafter, while there is apparently no increasing trend in the temporal evolution of area under drought under the more optimistic RCP 2.6 scenario. Based on the climate model simulation results, we find a seven-fold increase in the number of the 2-year drought events, covering at-least one third of Central European domain, in the second half of the century under the RCP 8.5 scenario as compared to the HistNat runs (inset of Fig. 3a). As a result, the corresponding fraction of attributable risk (FAR^40^; see Methods for more details) under RCP 8.5 is estimated to be nearly one, ascertaining a very strong anthropogenic contribution to exacerbating the occurrence probability of such drought events in the projected future period 2051–2100. Compared to the RCP 8.5 scenario, the number of 2-year droughts events reduces significantly by almost half under the RCP 4.5 scenario and to a very negligible number in RCP 2.6 for the projected period 2051–2100. The corresponding FAR values also reduce to 0.87 and 0.40 for the RCPs 4.5 and 2.6, respectively. These results clearly highlight the diverse role of anthropogenic activities in exacerbating the future occurrence of 2-year drought events; as well the possible benefits of mitigating measures to reduce carbon emissions (encoded as in RCP 2.6/4.5) in lowering the risk of the occurrence of consecutive drought events.

Consistent with the previous studies^8,10,41^, our analysis also shows that anthropogenic warming will lead to an intensification of European droughts, and to a large extent on the occurrence of 2-year droughts in the future. Such events have substantial implications on many sectors including impacts on agro-phenology, crop water demand and vegetation health activities. Using the long-term historical and projected land use changes based on HYDE database^42^ (see Methods), we find that drought affected cropland areas across the Central Europe will be nearly doubled (by $20 \pm 5$ million ha) under the RCP 8.5 scenario in the second half of the Century in comparison to corresponding historical values (Fig. 3b). This corresponds to the projection of nearly 60% of total cultivated areas being affected by drought in the Central Europe during 2051–2100. Adaptation strategies aiming at the amendment of global warming through the RCP 4.5/2.6 scenarios would significantly reduce the drought prone areas by almost 37%/60%, compared to RCP 8.5. A similar range of benefits in reducing the potential impacts of consecutive year droughts can be expected for areas covered with pastures (Fig. 3c)—which are of high importance for sustaining livestock (i.e., grazing).

## Conclusions

The present study analyses the occurrence of the consecutive droughts over the Central Europe in both historical and projected climate scenarios. The observational record suggests that the ongoing 2018–2019 European drought event is unprecedented in the last 250 years, with substantial implications for vegetation health. Our analysis based on an ensemble of climate model simulations suggests a strong increase in the occurrence of such a rare event, post 2050 under RCP 8.5 scenario. The frequency and the areal extent of these droughts strongly depend on the level of anthropogenic warming scenarios (as encoded in RCPs). Our analysis therefore demonstrates that the occurrences of the consecutive droughts as well their impact on crop and pasture areas can be significantly reduced, if the mitigation strategies leading to amendment of global warming are adopted. One of the major limitations of climate model simulation is its ability to reliably simulate the extreme events and the changes thereof^43^. Over Central Europe, we notice a general consensus between observations and climate models, especially post-1970, when the anthropogenic influences are apparent (Supplementary Fig. S5). Although, climate models have a relatively good ability to simulate historical past, larger uncertainties may still exist in projections^44^. Despite this limitation, the climate models are the only available tool to mechanistically understand the occurrence, processes and fate of future extreme events. Our study has mainly focused on detection and the future occurrence of the consecutive drought events. Although we show that under the increased global warming, the observed 2018–2019 droughts are going to increase in the future, an in-depth and separate (careful) analysis is required towards attributing the role of anthropogenic warming in modulating the occurrence of consecutive drought events. Further research is also needed to systematically understand driving mechanisms responsible for such consecutive droughts, whose value to climate adaptation can hardly be overemphasised.

## Methods

### Data

The assessment of consecutive drought characteristics from 1766–2019 is performed over the central Europe using three types of observed gridded meteorologic datasets: Casty et al^22^ for period 1766–1900, CRU TS dataset^23^ for period 1901–1949; and E-OBS^45^ for period 1950–2019. The composite dataset using monthly precipitation and air temperature is analysed at a spatial resolution of $0.5^\circ \, \times 0.5^\circ$, similarly as in to previous studies^25,46^. Furthermore, the E-OBS is used for correcting possible biases in the Casty and CRU data, which is trained on the overlapping period 1950–2015^25,46^. To examine the characteristics of the consecutive droughts in the past and future, we procure the state-of-the-art global climate model simulations from the Coupled Model Intercomparison Project phase 5 (CMIP5)^38^ (detailed description is provided in Supplementary Table S1). To quantify the effect of human activities in the past (1850–2005), two types of monthly forcings are analysed: (1) natural-only (HistNat), and (2) historical (Hist). While the HistNat contains only the effects of natural forcing (e.g., changes in solar radiation, volcanic eruptions), the Hist considers both, natural and anthropogenic (i.e., greenhouse gas concentrations) effects. To assess possible future climate scenarios, we procure three Representative Concentration Pathway (RCP) scenarios (2.6, 4.5 and 8.5), which are available for 2006–2100. Here, we select 11 climate models based on the consistency in data availability along with the parity in the climate variables (precipitation, temperature and net-radiation) used to estimate SPEI across all the simulations (HistNat, Hist and RCP scenarios). Compared to observations, the climate model simulations were able to capture the overall trend and patterns of atmospheric demand, particularly post-1970–the period when the human influence on the global warming is relatively more apparent^39^ (Supplementary Fig. S5). In this study, we did not apply bias corrections to the CMIP5 simulations. This is because the employed quantile-based SPEI estimates already account for systematic biases, particularly in the mean and standard deviation, as long as these do not lead to unrealistic P-E dynamics^47^, which is fairly well captured by climate model simulations (Supplementary Fig. S5).

The vegetation health index (VHI) is one of the important proxies, which is frequently used to evaluate the impacts of drought on vegetation health^48,49^. This index is applicable for assessing the vegetation stress and to examine the vegetation response to the natural hazards, such in our case, drought^49^. The VHI for the summer months is obtained from remote sensing data-sets^18^ at a weekly time step, where it is measured in percentile ranging from 0 to 100. A high value of VHI indicates healthy or unstressed vegetation condition, implying that these areas are not affected by drought conditions (i.e., lack of moisture conditions). The VHI of more than 50% shows above normal and/or healthy vegetation condition. Further, values ranging from 30 to 50% imply vegetation in the region suffering from moderate drought, and the VHI values less then 30% indicate a region experiencing severe drought leading to poor vegetation health conditions. In the present study, we considered VHI values $\le 30\%$ as a proxy for poor vegetation health conditions. Subsequently, we inferred the drought-affected vegetation activities as percentage of Central European area (shown in a rectangular box in Fig. 1g) exhibiting VHI $\le 30\%$.

### Drought analysis

Recent studies^24,50–53^ show the better performance of summer standardised precipitation evapotranspiration index (SPEI)^24,54,55^ in capturing the drought impacts on hydrological, ecological and agricultural variables than the standard precipitation index (SPI) or the Palmer drought severity index (PDSI). The SPI is not particularly appropriate for our application where both temperature and precipitation are important, since it neither considers the effects of increasing temperature over the recent decades^6,56^, nor the much larger warming scenario which is expected under future climate change scenarios^7^. Therefore, the characteristics of drought during summer months (June-August) in Central Europe for both observations and climate model simulations are estimated using SPEI. We used the non-parametric kernel-based approach to estimate the SPEI that can efficiently handle the multi-modality of the sample dataset as compared to other traditional parametric distributions^8,57^; and it can be represented as:

1 $$\begin{aligned} {\mathrm {SPEI}}=F_{t}(x_{t}) \end{aligned}$$

where, $x_{t}$ denotes the difference between precipitation (*P*) and potential evapotranspiration ($E_{p}$) at a time *t*. $F_{t}$ is the cumulative distribution function estimated using the kernel distribution $f_{t}(x)$ of the corresponding time *t*. $f_{t}(x)$ is estimated as

2 $$\begin{aligned} f_{t}(x)=\frac{1}{nh}\sum _{t=1}^{n}K \left( \frac{x-x_{t}}{h}\right) \end{aligned}$$

where *K* represents a Gaussian kernel function with a bandwidth *h*. The *h* is estimated by the Silverman approach^58^ for each grid cell separately. The SPEI value using the above-mentioned non-parametric approach varies between 0 and 1, with values below 0.5 indicate drier conditions and above 0.5 the wet conditions. A grid cell in central European region at time *t* is considered to be in drought when ${\hbox {SPEI}}_{t} \, \le \, \tau$. Here, $\tau$ denotes that the SPEI in the particular grid cell is less than the values occurring $\tau \, \times 100\%$ of the time, and the present study considers $\tau$ as 0.1 (i.e., 1 in 10-year event or 20% of all dry events)—indicating the occurrence of severe drought event^8,59^. In case of climate model simulation we also use the non-parametric kernel density estimator, however, we fix the bandwidth with respect to natural-forced historical simulation and use the same for historical; and for all the RCP scenarios considered in the present study. We estimated the yearly development of drought area ($A_t$), considering all the cells of the total Central European region that are under drought ($\hbox {SPEI} \le 0.1$) for a given year (*t*). We marked a drought event as a 2-year consecutive event when $A_t$ in both years crosses a certain threshold value (e.g., 33.3% or one-third of the Central European region). While estimating the number of consecutive drought events, especially in the RCP 4.5 and 8.5 scenarios, we notice many events with a common (overlapping) drought year. To account for the double counting effect, we counted those events as half which have an overlapping drought year between two (consecutive) events.

Considering the availability of climate variables over long time period (1766–2019), we estimate the monthly potential evapotranspiration ($E_{p}$) based from the mean temperature and the approximations for extraterrestrial solar radiation^60^. Owing to the limitation on the estimation the temperature based $E_{p}$^61^, we check the consistency of this method with an alternative and more physically based $E_p$ formulation. In this respect we use two $E_{p}$ datasets derived based on the Penman–Monteith method using: (a) the CRU database^23^ employing the mean, minimum and maximum temperature, vapour pressure, cloudiness and monthly climatology of wind speed available after 1901; and (b) the Princeton Global Forcing (PGF)^61^ that employs full scale variability of all required meteorological variables (e.g., net radiation, temperature and wind-speed) provided by Sheffield et al.^61^ for the period 1948–2008. Albeit different underlying meteorological databases being used (CRU^23^ vs. PGF^61^), in general, we notice a relatively good agreement among the three $E_{p}$ values, especially in capturing the inter-annual variability over the Central European region (see Supplementary Figs. S6 for more details).

We further check the consistency of our results based on the $E_{p}$ estimates derived from an energy budget approach following Milley and Dunne^62^, as given by:

3 $$\begin{aligned} E_{p} = 0.8 (R_{n} - G) \end{aligned}$$

where $R_{n}$ is net radiation at the surface, and *G* is ground heat flux. Here $R_{n} - G$ are estimated using the energy balance as: $R_{n} - G = L_{v}E + H$, where $L_{v}E$ and *H* are the latent and sensible heat flux, respectively. Using the climate model simulation outputs, our results show a high correspondence of $E_{p}$ between the energy-based approach^62^ and the Oudin et al.^60^ for the study domain (see Supplementary Fig. S7a,b for more details). Furthermore, the robustness of our findings on the increased occurrence of the future 2-year consecutive droughts is confirmed, regardless of the employed $E_{p}$ methods (see Supplementary Fig. S7c).

### Fraction of attributable risk (FAR)

The FAR has been used by many studies to quantify the anthropogenic influence on the occurrence of recent extreme events and its fate in projected scenarios. The FAR basically addresses the question of what fraction of extremes (in our case 2-year consecutive drought) occurring in Central European region is attributable to anthropogenic influence, and is given by,

4 $$\begin{aligned} FAR = 1 - (P_{0}/P_{1}) \end{aligned}$$

where $P_{0}$ is the probability of exceeding a 2-year consecutive drought without anthropogenic influence (HistNat) and $P_{1}$ is with the anthropogenic influence^40^ (RCP scenarios). The FAR value near to 1 indicates the nearly certain human influence in causing the 2-year consecutive drought.

### Cropland and pasture areas

The impact of droughts on cropland area and pastures are analysed using the dynamics of land use changes of land cover dataset^42^. This dataset consists of half-degree gridded historical and future fractional land-use patterns and underlying land-use transitions. The historical data uses the HYDE v3.1 historical data set for crop, pasture, and urban area 1500–2005, and the future land cover scenarios 2006–2100 are available for four Integrated Assessment Model (IAM) scenarios which reach different levels of radiative forcing by year 2100:, viz., MESSAGE (RCP 8.5), AIM (RCP 6.0), GCAM/minicam (RCP 4.5) and IMAGE (RCP 2.6). Further, each of these future projections are built by four different historical land-use products, all these are considered in our study.

The cropland cover fraction over the Central Europe started to increase during post 1950, however, a drastic decrease in spatial extent happened after 1990 (Supplementary Fig. S8a). In RCP 4.5 and RCP 8.5 scenarios, the Central Europe will experience a sharp decrease in the overall cropland area. This information is then combined with the fraction of total Central European area which is affected by droughts, as obtained from climate models (Supplementary Fig. S8c). We notice a prominent increasing trend of cropland area affected by drought, especially in the RCP 8.5 scenario. Similar behaviour is projected for the pastures as well (Supplementary Fig. S8b,d). With these observations, we notice a sharp increase in the areal effects both in cropland and pasture by the 2-year consecutive drought in the future, as shown in Fig. 3b. These findings remained same even when we considered a fixed, not time varying, area corresponding to the year 2005 (Supplementary Fig. S8e,f).

## Supplementary Information

Supplementary Information 1.

## Data Availability

Reconstructed historical precipitation and temperature (1766–1900) are available at ftp://ftp.ncdc.noaa.gov/pub/data/paleo/historical/europe/casty2007/. The HadCRU TS product (1901–1950 for precipitation, temperature, potential evapotranspiration) is available from http://catalogue.ceda.ac.uk/uuid/edf8febfdaad48abb2cbaf7d7e846a86. The E-OBS data (1951–2019) are available from https://www.ecad.eu/download/ensembles/download.php, the CMIP5 data from https://esgf-node.llnl.gov/projects/cmip5/, the VHI data from https://www.star.nesdis.noaa.gov/smcd/emb/vci/VH/vh_browse.php, and HYDE (v3.1) landcover data from https://themasites.pbl.nl/tridion/en/themasites/hyde/download/index-2.html . Other processed datasets can be made available upon reasonable request from the corresponding author.

## Code availability

The codes for estimating the SPEI based on kernel density approach can be acquired from PYTHON repository. Other processing codes can be procured from VH.

## References

[1] Pall P (2011). Anthropogenic greenhouse gas contribution to flood risk in England and Wales in autumn 2000. Nature.

[2] Min S-K, Zhang X, Zwiers FW, Hegerl GC (2011). Human contribution to more-intense precipitation extremes. Nature.

[3] Ciais P (2005). Europe-wide reduction in primary productivity caused by the heat and drought in 2003. Nature.

[4] Liu, X., He, B., Guo, L., Huang, L. & Chen, D. Similarities and differences in the mechanisms causing the European summer heatwaves in 2003, 2010, and 2018. *Earth’s Future***in press** (2020).

[5] Ionita M (2017). The European 2015 drought from a climatological perspective. Hydrol. Earth Syst. Sci..

[6] Luterbacher J, Dietrich D, Xoplaki E, Grosjean M, Wanner H (2004). European seasonal and annual temperature variability, trends, and extremes since 1500. Science.

[7] Barriopedro D, Fischer EM, Luterbacher J, Trigo RM, García-Herrera R (2011). The hot summer of 2010: redrawing the temperature record map of Europe. Science.

[8] Samaniego L (2018). Anthropogenic warming exacerbates European soil moisture droughts. Nat. Clim. Change.

[9] Dai A (2013). Increasing drought under global warming in observations and models. Nat. Clim. Change.

[10] Trenberth KE (2014). Global warming and changes in drought. Nat. Clim. Change.

[11] Huang J, Yu H, Guan X, Wang G, Guo R (2016). Accelerated dryland expansion under climate change. Nat. Clim. Change.

[12] He B (2018). Recovery of ecosystem carbon and energy fluxes from the 2003 drought in Europe and the 2012 drought in the United States. Geophys. Res. Lett..

[13] Scott RL, Jenerette GD, Potts DL, Huxman TE (2009). Effects of seasonal drought on net carbon dioxide exchange from a woody-plant-encroached semiarid grassland. J. Geophys. Res. Biogeosci..

[14] Pei F, Li X, Liu X, Lao C (2013). Assessing the impacts of droughts on net primary productivity in China. J. Environ. Manag..

[15] Thuiller W, Lavorel S, Araújo MB, Sykes MT, Prentice IC (2005). Climate change threats to plant diversity in europe. Proc. Nat. Acad. Sci..

[16] Brisson N (2010). Why are wheat yields stagnating in Europe? A comprehensive data analysis for France. Field Crops Res..

[17] Hawkins E (2013). Increasing influence of heat stress on french maize yields from the 1960s to the 2030s. Glob. Change Biol..

[18] Kogan FN (2001). Operational space technology for global vegetation assessment. Bull. Am. Meteorol. Soc..

[19] Toreti, A. *et al.* The exceptional 2018 European water seesaw calls for action on adaptation. *Earth’s Future* **7**, 652–663 (2019).

[20] Orth R, Zscheischler J, Seneviratne SI (2016). Record dry summer in 2015 challenges precipitation projections in central Europe. Sci. Rep..

[21] de Ruiter, M. C. *et al.* Why we can no longer ignore consecutive disasters. *Earth’s Future* **8**, e2019EF001425 (2019).

[22] Casty C, Raible CC, Stocker TF, Wanner H, Luterbacher J (2007). A European pattern climatology 1766–2000. Clim. Dyn..

[23] Harris I, Jones PD, Osborn TJ, Lister DH (2014). Updated high-resolution grids of monthly climatic observations-the CRU TS3. 10 Dataset. Int. J. Climatol..

[24] Vicente-Serrano SM, Beguería S, López-Moreno JI (2010). A multiscalar drought index sensitive to global warming: the standardized precipitation evapotranspiration index. J. Clim..

[25] Hanel M (2018). Revisiting the recent European droughts from a long-term perspective. Sci. Rep..

[26] Brunner L, Schaller N, Anstey J, Sillmann J, Steiner AK (2018). Dependence of present and future european temperature extremes on the location of atmospheric blocking. Geophys. Res. Lett..

[27] Brunner L, Hegerl GC, Steiner AK (2017). Connecting atmospheric blocking to European temperature extremes in spring. J. Clim..

[28] Miralles DG, Teuling AJ, Van Heerwaarden CC, De Arellano JV-G (2014). Mega-heatwave temperatures due to combined soil desiccation and atmospheric heat accumulation. Nat. Geosci..

[29] Zhou S (2019). Land–atmosphere feedbacks exacerbate concurrent soil drought and atmospheric aridity. Proc. Nat. Acad. Sci..

[30] Francis J, Skific N (2015). Evidence linking rapid Arctic warming to mid-latitude weather patterns. Philos. Trans. R. Soc. A Math., Phys. Eng. Sci..

[31] Francis JA, Vavrus SJ, Cohen J (2017). Amplified arctic warming and mid-latitude weather: new perspectives on emerging connections. Wiley Interdiscip. Rev. Clim. Change.

[32] Cohen J (2014). Recent Arctic amplification and extreme mid-latitude weather. Nat. Geosci..

[33] Zhang R, Sun C, Zhu J, Zhang R, Li W (2020). Increased European heat waves in recent decades in response to shrinking Arctic sea ice and Eurasian snow cover. npj Clim. Atmos. Sci..

[34] Francis JA, Vavrus SJ (2012). Evidence linking arctic amplification to extreme weather in mid-latitudes. Geophys. Res. Lett..

[35] Dai A, Luo D, Song M, Liu J (2019). Arctic amplification is caused by sea-ice loss under increasing CO2. Nat. Commun..

[36] Barnes EA, Polvani LM (2015). CMIP5 projections of Arctic amplification, of the North American/North Atlantic circulation, and of their relationship. J. Clim..

[37] Dai A, Song M (2020). Little influence of Arctic amplification on mid-latitude climate. Nat. Climate Change.

[38] Taylor KE, Stouffer RJ, Meehl GA (2012). An overview of CMIP5 and the experiment design. Bull. Am. Meteorol. Soc..

[39] Diffenbaugh NS, Swain DL, Touma D (2015). Anthropogenic warming has increased drought risk in California. Proc. Nat. Acad. Sci..

[40] Fischer EM, Knutti R (2015). Anthropogenic contribution to global occurrence of heavy-precipitation and high-temperature extremes. Nat. Clim. Change.

[41] Lehner F (2017). Projected drought risk in 1.5 C and 2 C warmer climates. Geophys. Res. Lett..

[42] Klein Goldewijk K, Beusen A, Van Drecht G, De Vos M (2011). The HYDE 3.1 spatially explicit database of human-induced global land-use change over the past 12,000 years. Glob. Ecol. Biogeogr..

[43] Bellprat O, Guemas V, Doblas-Reyes F, Donat MG (2019). Towards reliable extreme weather and climate event attribution. Nat. Commun..

[44] James R, Washington R, Schleussner C-F, Rogelj J, Conway D (2017). Characterizing half-a-degree difference: a review of methods for identifying regional climate responses to global warming targets. Wiley Interdiscip. Rev. Clim. Change.

[45] Hofstra N, Haylock M, New M, Jones PD (2009). Testing E-OBS European high-resolution gridded data set of daily precipitation and surface temperature. J. Geophys. Res. Atmos..

[46] Moravec V, Markonis Y, Rakovec O, Kumar R, Hanel M (2019). A 250-year European drought inventory derived from ensemble hydrologic modeling. Geophys. Res. Lett..

[47] Thober S (2015). Seasonal soil moisture drought prediction over Europe using the North American multi-model ensemble (NMME). J. Hydrometeorol..

[48] Kogan FN (1997). Global drought watch from space. Bull. Am. Meteorol. Soc..

[49] Bachmair S, Tanguy M, Hannaford J, Stahl K (2018). How well do meteorological indicators represent agricultural and forest drought across Europe?. Environ. Res. Lett..

[50] Marcos R, Turco M, Bedía J, Llasat MC, Provenzale A (2015). Seasonal predictability of summer fires in a mediterranean environment. Int. J. Wildl. Fire.

[51] Turco M, Levin N, Tessler N, Saaroni H (2017). Recent changes and relations among drought, vegetation and wildfires in the Eastern Mediterranean: the case of Israel. Global Planet. Change.

[52] Labudová L, Labuda M, Takáč J (2017). Comparison of SPI and SPEI applicability for drought impact assessment on crop production in the Danubian Lowland and the East Slovakian Lowland. Theoret. Appl. Climatol..

[53] Turco M (2017). On the key role of droughts in the dynamics of summer fires in Mediterranean Europe. Sci. Rep..

[54] Stagge JH, Kingston DG, Tallaksen LM, Hannah DM (2017). Observed drought indices show increasing divergence across Europe. Sci. Rep..

[55] Vicente-Serrano SM, McVicar TR, Miralles DG, Yang Y, Tomas-Burguera M (2019). Unraveling the influence of atmospheric evaporative demand on drought and its response to climate change. Wiley Interdiscip. Rev. Clim. Change.

[56] Schär C (2004). The role of increasing temperature variability in European summer heatwaves. Nature.

[57] Adamowski K (1996). Nonparametric estimation of low-flow frequencies. J. Hydraul. Eng..

[58] Silverman BW (1986). Density Estimation for Statistics and Data Analysis.

[59] Sheffield J, Wood EF (2008). Global trends and variability in soil moisture and drought characteristics, 1950–2000, from observation-driven simulations of the terrestrial hydrologic cycle. J. Clim..

[60] Oudin L (2005). Which potential evapotranspiration input for a lumped rainfall-runoff model?: Part 2-towards a simple and efficient potential evapotranspiration model for rainfall-runoff modelling. J. Hydrol..

[61] Sheffield J, Wood EF, Roderick ML (2012). Little change in global drought over the past 60 years. Nature.

[62] Milly PC, Dunne KA (2016). Potential evapotranspiration and continental drying. Nat. Clim. Change.

