# Supplementary material for: Increased future occurrences of the exceptional 2018–2019 Central European drought under global warming
Source: Sci Rep. 2020 Aug 6;10:12207. doi: 10.1038/s41598-020-68872-9 (PMC7413549; doi:10.1038/s41598-020-68872-9)
Supplement: Supplementary file 1 — Supplementary Information 1. [file 41598_2020_68872_MOESM1_ESM.pdf]

Supplementary Material for

Increased future occurrences of the exceptional 2018–2019 Central European drought under global warming

Vittal Hari[1,*], Oldrich Rakovec[1,2,*], Yannis Markonis[2], Martin Hanel[2], and Rohini Kumar[1,*]

[1]UFZ-Helmholtz Centre for Environmental Research, Leipzig, 04318, Germany

[2]Faculty of Environmental Sciences, Czech University of Life Sciences Prague, Kamýcká 129, Praha – Suchdol, 165 00, Czech Republic

[*]Authors with equal contributions

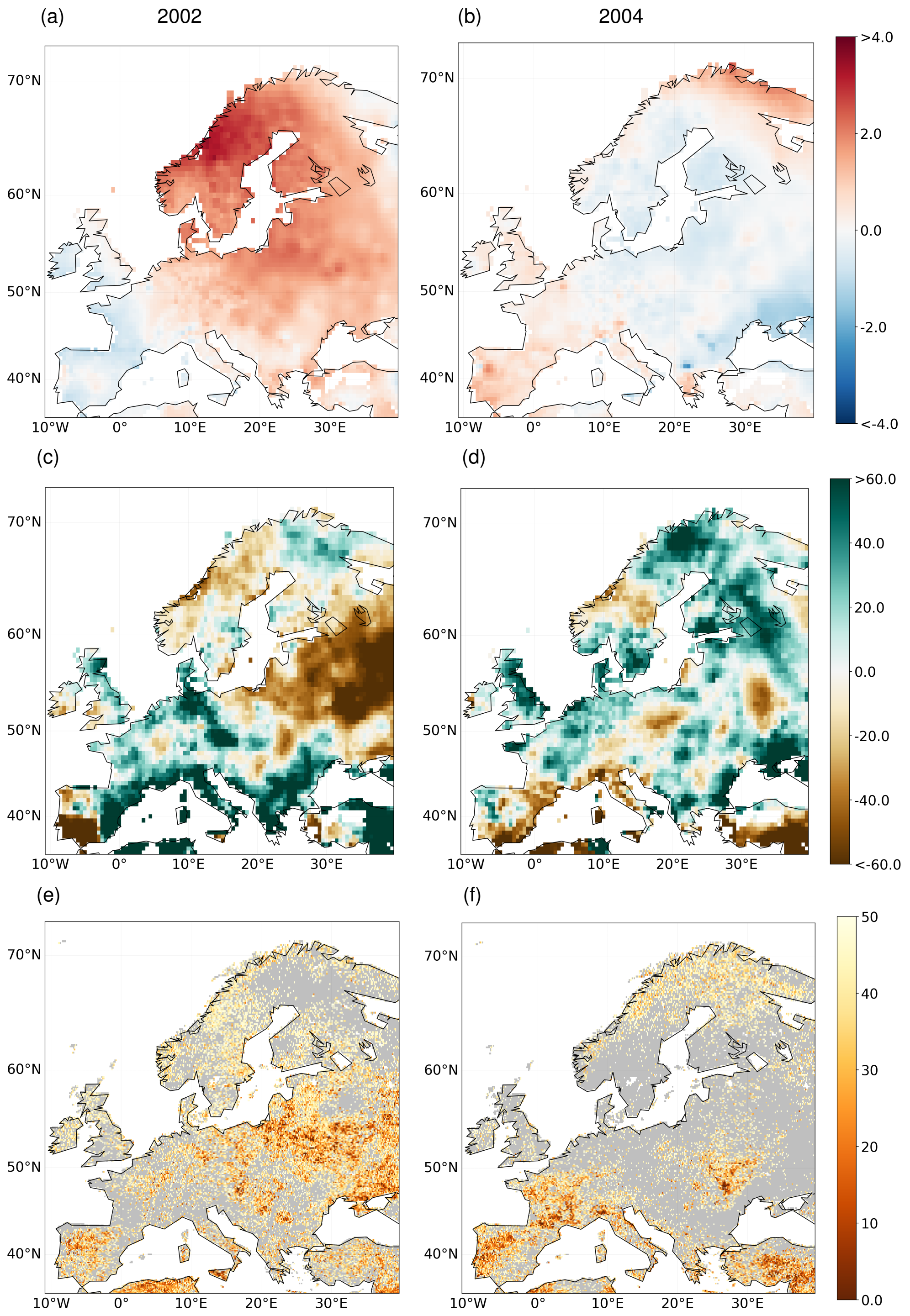

Figure S1: **Anomalies of climate and vegetation health index (VHI) during 2002 and 2004.** (a, b) Temperature anomaly (^{o}C) for 2002 and 2004 based on the 1980–2010 climatology. (c, d) Precipitation anomaly (%) for 2002 and 2004. (e, f) Vegetation condition in terms of VHI during 2002 and 2004. The maps in the figure are generated using Python version 3.7.3 (`https://www.python.org/search/?q=Python+3.7.3`).

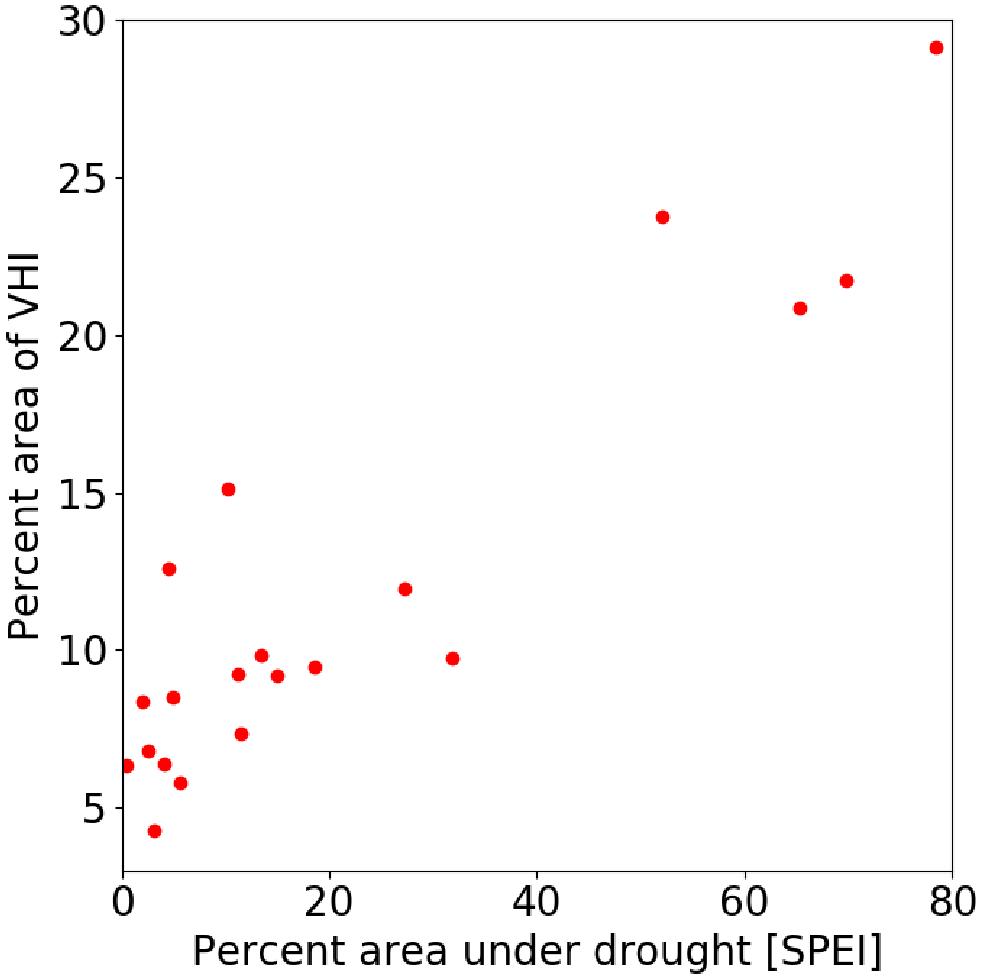

Figure S2: Relationship between percent area of drought estimated from summer SPEI and percent area of VHI ($\leq$ 30%) in Central Europe from 2000 to 2019. The correlation between these two variables is 0.62, which is significant at 5% significance level.

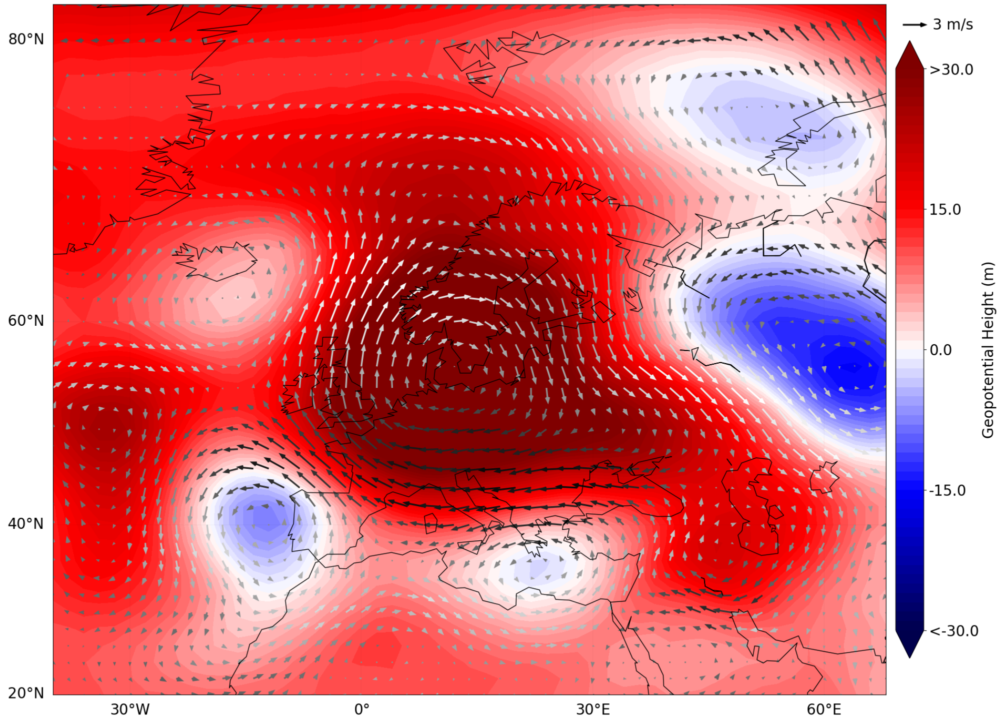

Figure S3: Characteristics of large-scale atmospheric circulation during 2018–2019 summer drought. The composite anomaly of geopotential height (shaded) and wind (vectors) at 500 hpa are represented here with the use of recently archived ERA5 reanalysis dataset[1]. The map in the figure is generated using Python version 3.7.3 (`https://www.python.org/search/?q=Python+3.7.3`).

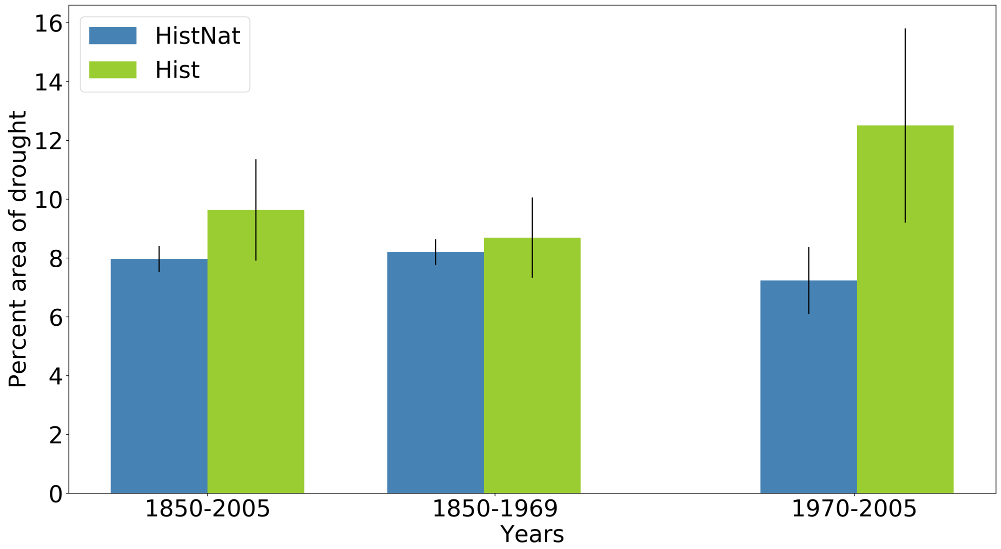

Figure S4: Differences in the temporal evolution of area under drought over the Central Europe for natural-only forced (HistNat) and historical (Hist) simulations during different time periods (whole: 1850-2005, and two subsets of 1850–1969 and 1970–2005). Barplots represent the ensemble mean of the areal extent of drought, whereas the error bars represents the 95% confidence limits based on the sampling distribution of the mean.

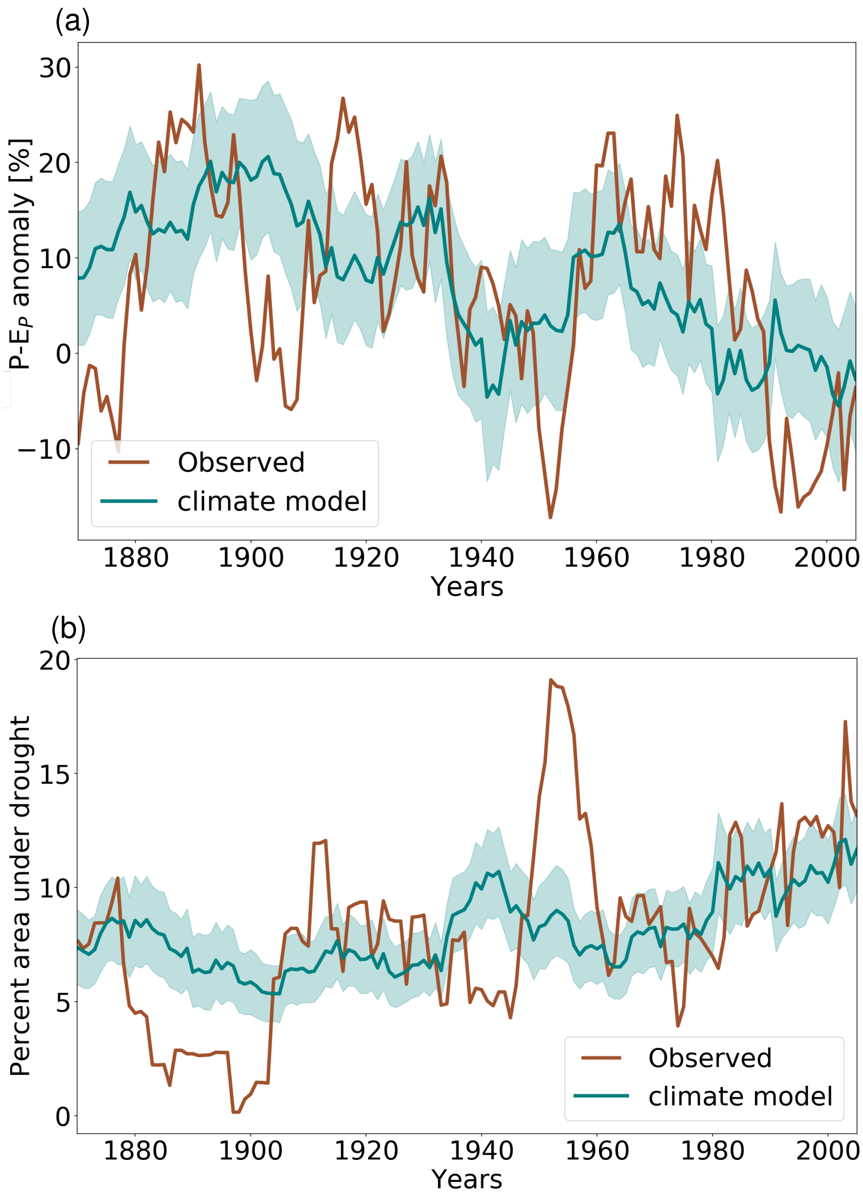

Figure S5: (a) Precipitation (P) minus potential evapotranspiration (E_p) anomaly during summer months (JJA) averaged over Central Europe based on CMIP5 all-forcings historical experiment (teal) and the observations (sienna) during 1860–2005, considering 10-year moving average. The anomaly is based on the 1970–2010 climatology. The thick teal lines show the multimodel means from 11 CMIP5 models considered in the study. The filled areas around each of the solid lines represent the 95% confidence intervals based on the sampling distribution of the mean. (b) is same as (a), but for percent areal extent of drought over Central Europe. We notice a consistency of climate model simulation with observed patterns and trends, specifically post-1970.

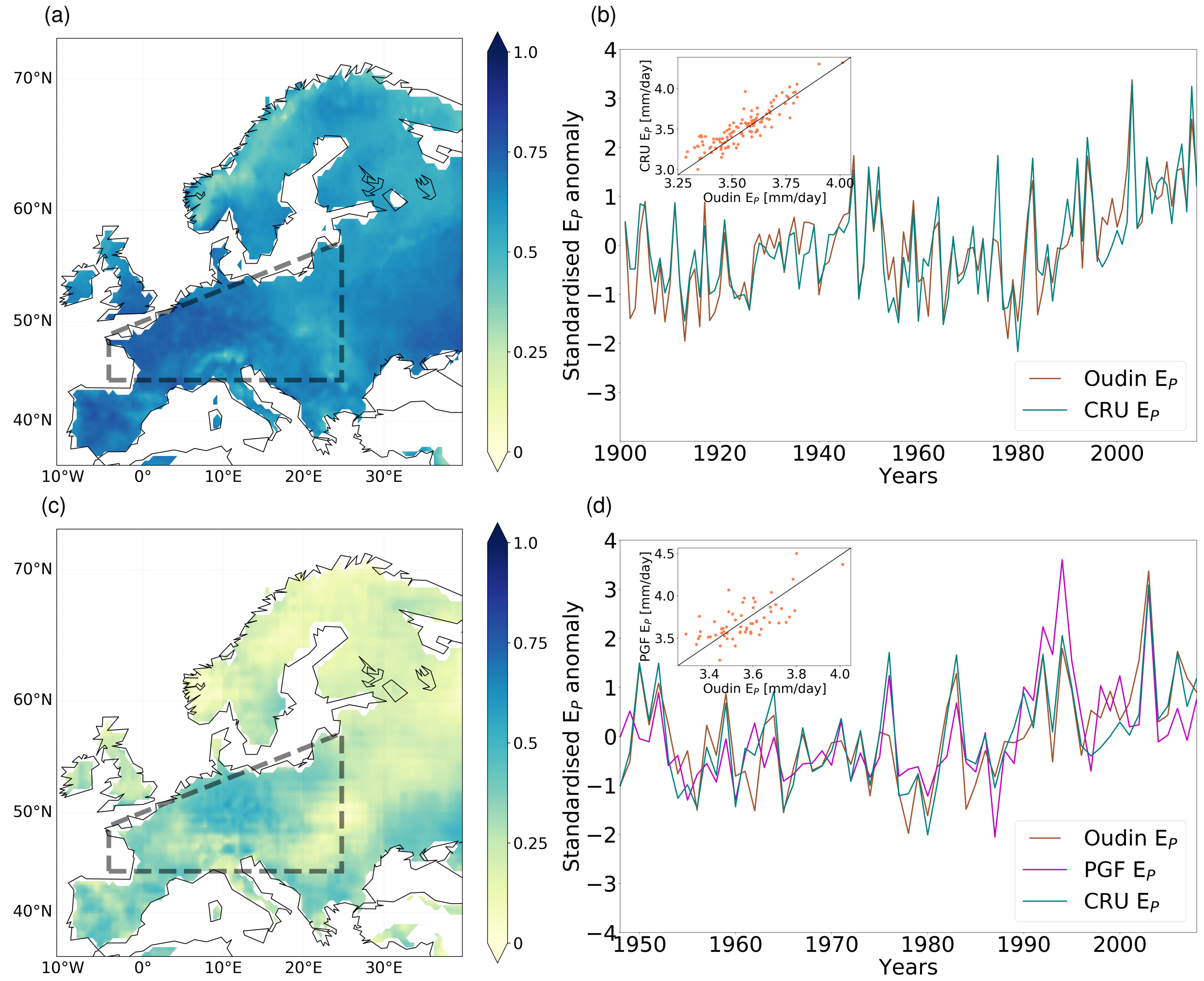

Figure S6: (a) Correlation between summer (JJA) potential evapotranspiration (E_p) estimates based on extraterrestrial solar radiation (Oudin)[2] and CRU-based Penman-Monteith method[3]. (b) Temporal evolution of standardised E_p anomaly from 1901–2016 over the Central European region (depicted by a black rectangular region in a). The inset of (b) represents the relationship between Oudin and CRU-based E_p over the Central European region. (c) Correlation between summer (JJA) E_p estimates based on Oudin method and Princeton Global Forcings (PGF) based E_p method[4] using full scale variability of climate variables (including net radiation, temperature and wind-speed) for the common period 1948–2008. The presented E_p anomaly in a standardised form is relevant for capturing the inter-annual variability as deemed for the drought index based on SPEI. (d) is same as (b), but also with PGF based E_p for the common period 1948–2008. Despite the differences in underlying meteorological databases (CRU[3] vs. PGF[4]), we find overall a reasonably good agreement between different E_p datasets, especially in capturing the inter-annual variability over the Central European region as depicted in the panels (b) and (d). The maps in the figure are generated using Python version 3.7.3 (`https://www.python.org/search/?q=Python+3.7.3`).

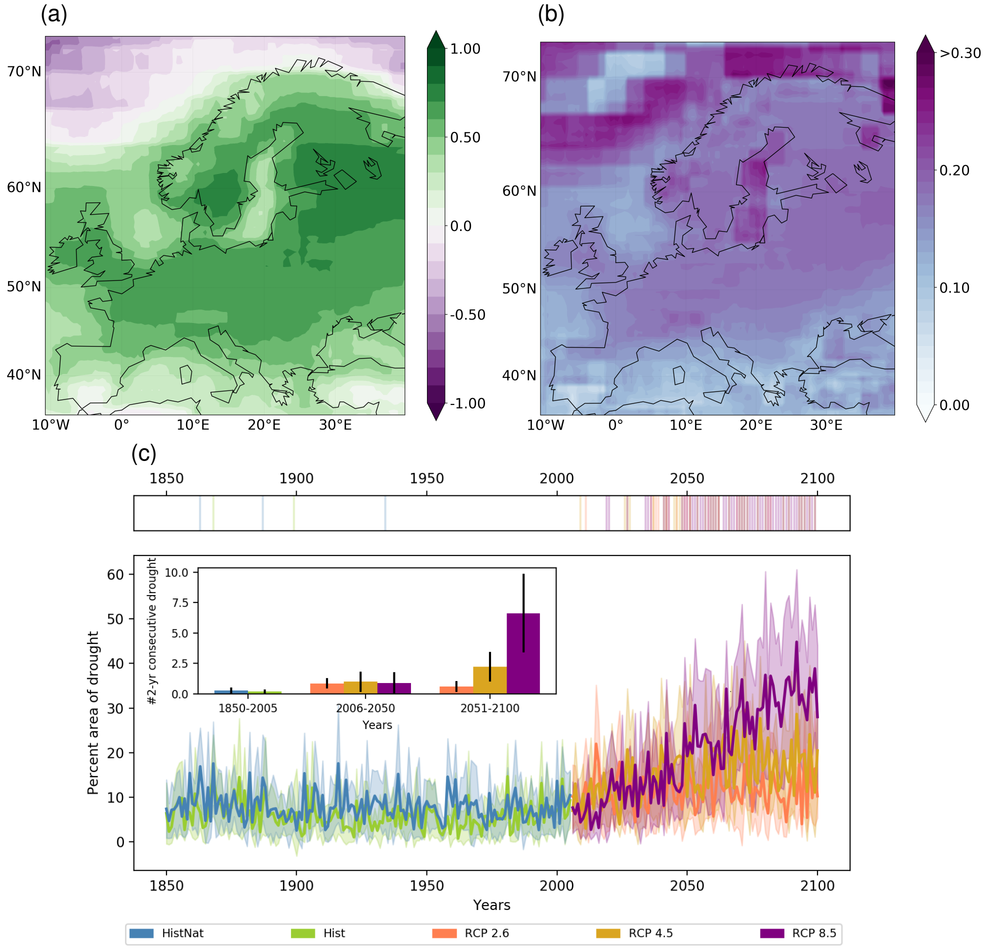

Figure S7: (a) Relationship between summer temperature-based E_p with the net radiation (R_n) approach, (see equation 3 in the main text) based on naturally-forced climate model historical simulation (HistNat) from 1850 to 2005. Here we show the ensemble mean of correlation between these two variables from the 11 considered climate models, whereas its standard deviation is presented in (b). (c) Yearly development of the percent area of drought over Central Europe based on the ensemble (N=11) of climate model simulations from CMIP5 under different experimental scenarios: natural only historical (HistNat), all-forcings historical (Hist), and three future RCPs (2.6, 4.5, and 8.5). E_p for this analysis is estimated using equation (3) from the main text. The thick solid lines show the multimodel means, and the filled areas represent the 95% confidence intervals based on the sampling distribution of the mean. The inset plot in (c) represents the number of 2-year droughts, with an areal extent in each year covering at-least one third of the Central European region, estimated over the specified time-period for different experimental scenarios (i.e., 1850–2005 for the Hist and HistNat; and 2006–2050/2051–2100 for the RCPs). Shown are the ensemble mean and 95% confidence limits based on the sampling distribution of the mean, corresponding to the climate model outputs. The top panel of (c) depicts the year in which any of the 11 climate models show the 2-year droughts. The maps in the figure are generated using Python version 3.7.3 (`https://www.python.org/search/?q=Python+3.7.3`).

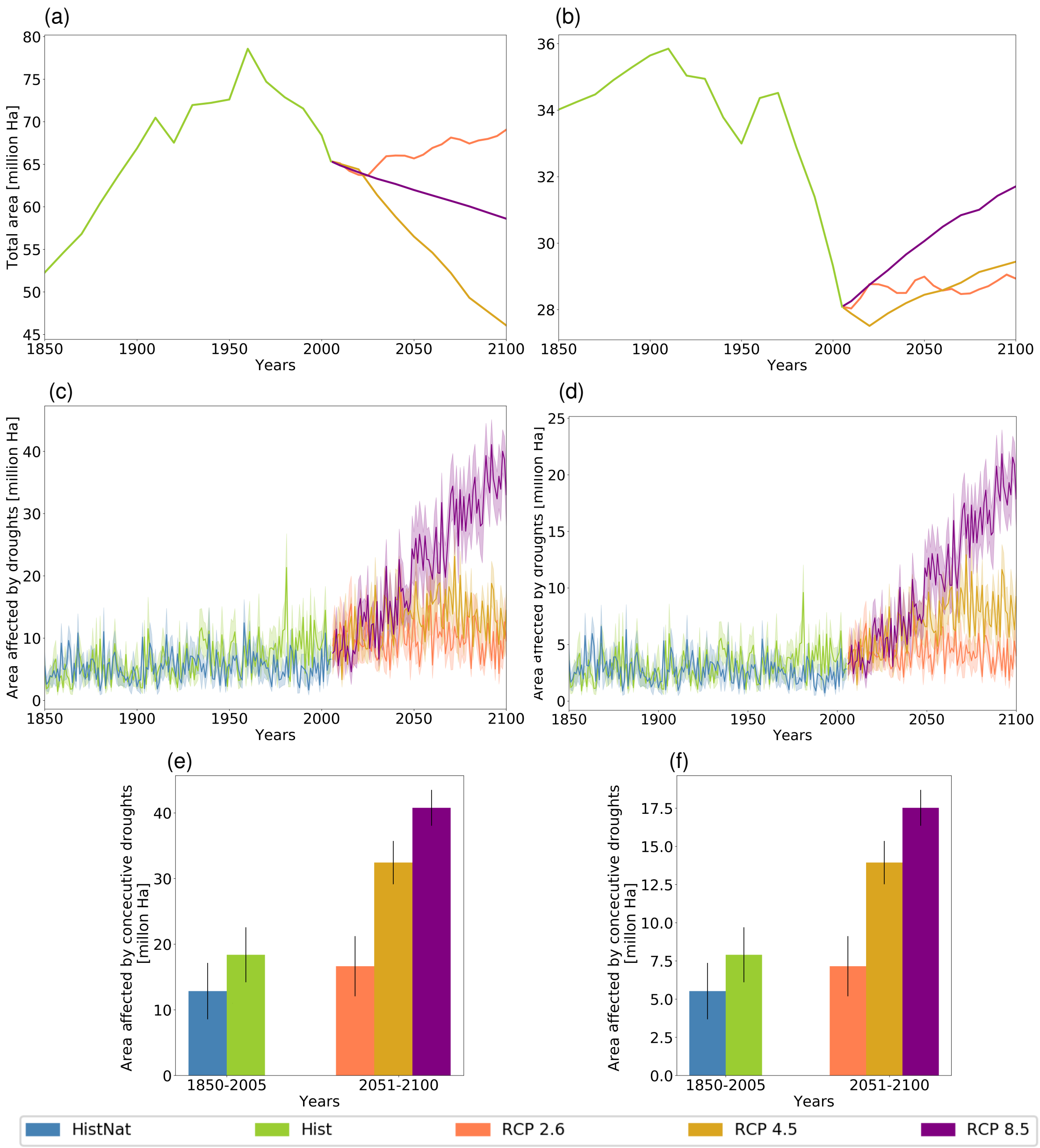

Figure S8: (a, b) Variation of crop land and pasture total areas in central Europe for past and different future RCP scenarios as obtained from HYDE 3.1 database. (c, d) Time series of fraction of area affected in million hectares by drought for crop and pasture land. The thick lines show the multimodel means from the climate models. The filled areas around each of the solid lines represent the 95% confidence intervals based on the sampling distribution of the mean. (e) Crop land area effected by 2-year droughts for different time periods, considering different scenarios of climate model experiment simulations. Here we fix the total area based on the simulation from HYDE 3.1 for the year 2005. (f) Same as (e), but for pasture land over central Europe.

Table S1: List of climate models used in the present study. The spatial resolution of the climate model simulations were converted to that of observed data (i.e., 0.5° × 0.5°) based on the nearest neighbour approach.

**Models**	**Spatial resolution**	**Organization**
CNRM–CM5	1.4° × 1.4°	Centre National de Recherches Météotologiques and Centre Européen de Recherche et Formation Abancées en Calcul Scientifique, France
FGOALS–g2	3° × 3°	LASG, Institute of Atmospheric Physics, Chinese Academy of Sciences, China
GFDL–CM3	2° × 2°	NOAA/Geophysical Fluid Dynamic Laboratory, United States
GISS–E2–H	2° × 2°	NASA Goddard Institute for Space Studies, United States
GISS–E2–R	2° × 2°	NASA Goddard Institute for Space Studies, United States
HadGEM2–ES	1.2° × 1.8°	Met Office Hadley Centre, United Kingdom
IPSL–CM5A–LR	2° × 4°	Laboratoire de Météotologic Dynamique and L'Instutut Pierre–Simon Laplace, France
IPSL–CM5A–MR	2° × 4°	Laboratoire de Météotologic Dynamique and L'Instutut Pierre–Simon Laplace, France
MIROC–ESM	3° × 3°	Atmosphere and Ocean Research Institute, National Institute for Environmental Studies, and Japan Agency for Marine–Earth Science and Technology, Japan
MIROC-ESM-CHEM	3° × 3°	Atmosphere and Ocean Research Institute, National Institute for Environmental Studies, and Japan Agency for Marine–Earth Science and Technology, Japan
MRI-CGCM3	1° × 1°	Meteorological Research Institute, Japan Meteorological Agency, Japan

1. Hersbach, H. *et al.* The era5 global reanalysis. *Quarterly Journal of the Royal Meteorological Society* **n/a** (2020 in press).

2. Oudin, L. *et al.* Which potential evapotranspiration input for a lumped rainfall–runoff model?: Part 2—Towards a simple and efficient potential evapotranspiration model for rainfall–runoff modelling. *Journal of Hydrology* **303**, 290–306 (2005).

3. Harris, I. c., Jones, P. D., Osborn, T. J. & Lister, D. H. Updated high-resolution grids of monthly climatic observations–the CRU TS3. 10 Dataset. *International Journal of Climatology* **34**, 623–642 (2014).

4. Sheffield, J., Wood, E. F. & Roderick, M. L. Little change in global drought over the past 60 years. *Nature* **491**, 435–438 (2012).
